# Hystological paraspinal muscle comparison between curve concavity and convexity in adolescent idiopathic scoliosis

**DOI:** 10.1186/1748-7161-9-S1-O12

**Published:** 2014-12-04

**Authors:** Marcelo Wajchenberg, Rafael Paiva Luciano, Delio Eulalio Martins, Luciano Miler Reis Rodrigues, Eduardo Barros Puertas, Moises Cohen, David Del Curto, Acary Souza Bulle de Oliveira, Beny Schmidt

**Affiliations:** 1 Federal University of Sao Paulo, Sao Paulo, Brazil; 2 ABC University, Sao Paulo, Brazil

## Background

Several studies have presented morphological, biochemical and histopathological changes in the paraspinal skeletal muscle of patients with adolescent idiopathic scoliosis (AIS). Some abnormalities have been demonstrated such as an increased amount of type I fibers in the concavity and the decreased number of type II in the convexity, an increase in the intracellular concentration of glycogen and lipids, structural changes in the sarcolemma and myotendinous junction, changes in the activity muscle enzyme, an increase in intracellular calcium concentration.

## Aim

To evaluate the rotator muscle fibers at the apical vertebraof curves of patients with AIS by histological and immunohistochemical analysis.

## Design

Cross-sectional study.

## Methods

Twenty-one patients with AIS submitted to surgical correction between 2,010 and 2,013 had the spinal rotator muscles biopsied in the concavity and convexity of the apical vertebra of the thoracic curve during the surgery. Serial cryosections were stained with Hematoxylin-Eosin (HE) and Sudan Red. We evaluated muscle atrophy and hypertrophy, fatty proliferation, presence of endomysial and perimysial fibrosis, presence of hyaline fibers, mitochondrial proliferation, muscle necrosis, nuclear centralization and inflammation. Two independent observers evaluated the sections.

## Results

The average value of the angle of the main thoracic curve was 68° Cobb. For analysis of non-parametric paired variables between the concave and convex side it was used the McNemar test with a significance level of 5%. The results of relative frequency and “p” values showed significant difference in both endomysial and perimysialfibrosis and fatty involution when compared to both sides of the apical vertebra.

## Conclusion

The paraspinal muscles at the apex of the deformity had higher involvement in the concavity when analyzing the fatty involution and fibrosis. However, both sides showed signs of myopathy, muscle atrophy through areas of necrosis and hyaline presence of fibers, mitochondrial proliferation.

